# Co-infections, secondary infections, and antimicrobial use in patients hospitalised with COVID-19 during the first pandemic wave from the ISARIC WHO CCP-UK study: a multicentre, prospective cohort study

**DOI:** 10.1016/S2666-5247(21)00090-2

**Published:** 2021-08

**Authors:** Clark D Russell, Cameron J Fairfield, Thomas M Drake, Lance Turtle, R Andrew Seaton, Dan G Wootton, Louise Sigfrid, Ewen M Harrison, Annemarie B Docherty, Thushan I de Silva, Conor Egan, Riinu Pius, Hayley E Hardwick, Laura Merson, Michelle Girvan, Jake Dunning, Jonathan S Nguyen-Van-Tam, Peter J M Openshaw, J Kenneth Baillie, Malcolm G Semple, Antonia Ho, ISARIC4C investigators, J Kenneth Baillie, Malcolm G Semple, Peter JM Openshaw, Gail Carson, Beatrice Alex, Benjamin Bach, Wendy S Barclay, Debby Bogaert, Meera Chand, Graham S Cooke, Annemarie B Docherty, Jake Dunning, Ana da Silva Filipe, Tom Fletcher, Christopher A Green, Ewen M Harrison, Julian A Hiscox, Antonia YW Ho, Peter W Horby, Samreen Ijaz, Say Khoo, Paul Klenerman, Andrew Law, Wei Shen Lim, Alexander J Mentzer, Laura Merson, Alison M Meynert, Mahdad Noursadeghi, Shona C Moore, Massimo Palmarini, William A Paxton, Georgios Pollakis, Nicholas Price, Andrew Rambaut, David L Robertson, Clark D Russell, Vanessa Sancho-Shimizu, Janet T Scott, Thushan de Silva, Louise Sigfrid, Tom Solomon, Shiranee Sriskandan, David Stuart, Charlotte Summers, Richard S Tedder, Emma C Thomson, AA Roger Thompson, Ryan S Thwaites, Lance CW Turtle, Rishi K Gupta, Carlo Palmieri, Maria Zambon, Hayley Hardwick, Chloe Donohue, Ruth Lyons, Fiona Griffiths, Wilna Oosthuyzen, Lisa Norman, Riinu Pius, Thomas M Drake, Cameron J Fairfield, Stephen R Knight, Kenneth A Mclean, Derek Murphy, Catherine A Shaw, Jo Dalton, Michelle Girvan, Egle Saviciute, Stephanie Roberts, Janet Harrison, Laura Marsh, Marie Connor, Sophie Halpin, Clare Jackson, Carrol Gamble, Gary Leeming, Andrew Law, Murray Wham, Sara Clohisey, Ross Hendry, James Scott-Brown, William Greenhalf, Victoria Shaw, Sarah E McDonald, Seán Keating, Katie A. Ahmed, Jane A Armstrong, Milton Ashworth, Innocent G Asiimwe, Siddharth Bakshi, Samantha L Barlow, Laura Booth, Benjamin Brennan, Katie Bullock, Benjamin WA Catterall, Jordan J Clark, Emily A Clarke, Sarah Cole, Louise Cooper, Helen Cox, Christopher Davis, Oslem Dincarslan, Chris Dunn, Philip Dyer, Angela Elliott, Anthony Evans, Lorna Finch, Lewis WS Fisher, Terry Foster, Isabel Garcia-Dorival, William Greenhalf, Philip Gunning, Catherine Hartley, Rebecca L Jensen, Christopher B Jones, Trevor R Jones, Shadia Khandaker, Katharine King, Robyn T. Kiy, Chrysa Koukorava, Annette Lake, Suzannah Lant, Diane Latawiec, Lara Lavelle-Langham, Daniella Lefteri, Lauren Lett, Lucia A Livoti, Maria Mancini, Sarah McDonald, Laurence McEvoy, John McLauchlan, Soeren Metelmann, Nahida S Miah, Joanna Middleton, Joyce Mitchell, Shona C Moore, Ellen G Murphy, Rebekah Penrice-Randal, Jack Pilgrim, Tessa Prince, Will Reynolds, P. Matthew Ridley, Debby Sales, Victoria E Shaw, Rebecca K Shears, Benjamin Small, Krishanthi S Subramaniam, Agnieska Szemiel, Aislynn Taggart, Jolanta Tanianis-Hughes, Jordan Thomas, Erwan Trochu, Libby van Tonder, Eve Wilcock, J. Eunice Zhang, Lisa Flaherty, Nicole Maziere, Emily Cass, Alejandra Doce Carracedo, Nicola Carlucci, Anthony Holmes, Hannah Massey, Lee Murphy, Nicola Wrobel, Sarah McCafferty, Kirstie Morrice, Alan MacLean, Kayode Adeniji, Daniel Agranoff, Ken Agwuh, Dhiraj Ail, Erin L. Aldera, Ana Alegria, Brian Angus, Abdul Ashish, Dougal Atkinson, Shahedal Bari, Gavin Barlow, Stella Barnass, Nicholas Barrett, Christopher Bassford, Sneha Basude, David Baxter, Michael Beadsworth, Jolanta Bernatoniene, John Berridge, Nicola Best, Pieter Bothma, David Chadwick, Robin Brittain-Long, Naomi Bulteel, Tom Burden, Andrew Burtenshaw, Vikki Caruth, David Chadwick, Duncan Chambler, Nigel Chee, Jenny Child, Srikanth Chukkambotla, Tom Clark, Paul Collini, Catherine Cosgrove, Jason Cupitt, Maria-Teresa Cutino-Moguel, Paul Dark, Chris Dawson, Samir Dervisevic, Phil Donnison, Sam Douthwaite, Ingrid DuRand, Ahilanadan Dushianthan, Tristan Dyer, Cariad Evans, Chi Eziefula, Chrisopher Fegan, Adam Finn, Duncan Fullerton, Sanjeev Garg, Sanjeev Garg, Atul Garg, Effrossyni Gkrania-Klotsas, Jo Godden, Arthur Goldsmith, Clive Graham, Elaine Hardy, Stuart Hartshorn, Daniel Harvey, Peter Havalda, Daniel B Hawcutt, Maria Hobrok, Luke Hodgson, Anil Hormis, Michael Jacobs, Susan Jain, Paul Jennings, Agilan Kaliappan, Vidya Kasipandian, Stephen Kegg, Michael Kelsey, Jason Kendall, Caroline Kerrison, Ian Kerslake, Oliver Koch, Gouri Koduri, George Koshy, Shondipon Laha, Steven Laird, Susan Larkin, Tamas Leiner, Patrick Lillie, James Limb, Vanessa Linnett, Jeff Little, Mark Lyttle, Michael MacMahon, Emily MacNaughton, Ravish Mankregod, Huw Masson, Elijah Matovu, Katherine McCullough, Ruth McEwen, Manjula Meda, Gary Mills, Jane Minton, Mariyam Mirfenderesky, Kavya Mohandas, Quen Mok, James Moon, Elinoor Moore, Patrick Morgan, Craig Morris, Katherine Mortimore, Samuel Moses, Mbiye Mpenge, Rohinton Mulla, Michael Murphy, Megan Nagel, Thapas Nagarajan, Mark Nelson, Matthew K. O'Shea, Igor Otahal, Marlies Ostermann, Mark Pais, Selva Panchatsharam, Danai Papakonstantino, Hassan Paraiso, Brij Patel, Natalie Pattison, Justin Pepperell, Mark Peters, Mandeep Phull, Stefania Pintus, Jagtur Singh Pooni, Frank Post, David Price, Rachel Prout, Nikolas Rae, Henrik Reschreiter, Tim Reynolds, Neil Richardson, Mark Roberts, Devender Roberts, Alistair Rose, Guy Rousseau, Brendan Ryan, Taranprit Saluja, Aarti Shah, Prad Shanmuga, Anil Sharma, Anna Shawcross, Jeremy Sizer, Manu Shankar-Hari, Richard Smith, Catherine Snelson, Nick Spittle, Nikki Staines, Tom Stambach, Richard Stewart, Pradeep Subudhi, Tamas Szakmany, Kate Tatham, Jo Thomas, Chris Thompson, Robert Thompson, Ascanio Tridente, Darell Tupper-Carey, Mary Twagira, Andrew Ustianowski, Nick Vallotton, Lisa Vincent-Smith, Shico Visuvanathan, Alan Vuylsteke, Sam Waddy, Rachel Wake, Andrew Walden, Ingeborg Welters, Tony Whitehouse, Paul Whittaker, Ashley Whittington, Padmasayee Papineni, Meme Wijesinghe, Martin Williams, Lawrence Wilson, Sarah Sarah, Stephen Winchester, Martin Wiselka, Adam Wolverson, Daniel G Wootton, Andrew Workman, Bryan Yates, Peter Young

**Affiliations:** a University of Edinburgh Centre for Inflammation Research, Edinburgh, UK; b Roslin Institute, University of Edinburgh, Easter Bush, Edinburgh, UK; c Centre for Medical Informatics, Usher Institute, University of Edinburgh, Edinburgh, UK; d Institute of Infection, Veterinary and Ecological Sciences, Faculty of Health and Life Sciences, University of Liverpool, Liverpool, UK; e Liverpool Clinical Trials Centre, University of Liverpool, Liverpool, UK; f Liverpool University Hospitals NHS Foundation Trust, Liverpool, UK; g Department of Infectious Diseases, Queen Elizabeth University Hospital, Glasgow, UK; h ISARIC Global Support Centre, Centre for Tropical Medicine and Global Health, Nuffield Department of Medicine, University of Oxford, Oxford, UK; i South Yorkshire Regional Department of Infection and Tropical Medicine, Sheffield Teaching Hospitals NHS Foundation Trust, Sheffield, UK; j Department of Infection, Immunity and Cardiovascular Disease, Medical School, University of Sheffield, UK; k Emerging Infections and Zoonoses Unit, National Infection Service, Public Health England, Colindale, London, UK; l Division of Epidemiology and Public Health, University of Nottingham School of Medicine, Nottingham, UK; m UK Department of Health and Social Care, London, UK; n National Heart and Lung Institute, Imperial College London, London, UK; o Department of Respiratory Medicine, Alder Hey Children's Hospital, Liverpool, UK; p Medical Research Council—University of Glasgow Centre for Virus Research, University of Glasgow, Glasgow, UK

## Abstract

**Background:**

Microbiological characterisation of co-infections and secondary infections in patients with COVID-19 is lacking, and antimicrobial use is high. We aimed to describe microbiologically confirmed co-infections and secondary infections, and antimicrobial use, in patients admitted to hospital with COVID-19.

**Methods:**

The International Severe Acute Respiratory and Emerging Infections Consortium (ISARIC) WHO Clinical Characterisation Protocol UK (CCP-UK) study is an ongoing, prospective cohort study recruiting inpatients from 260 hospitals in England, Scotland, and Wales, conducted by the ISARIC Coronavirus Clinical Characterisation Consortium. Patients with a confirmed or clinician-defined high likelihood of SARS-CoV-2 infection were eligible for inclusion in the ISARIC WHO CCP-UK study. For this specific study, we excluded patients with a recorded negative SARS-CoV-2 test result and those without a recorded outcome at 28 days after admission. Demographic, clinical, laboratory, therapeutic, and outcome data were collected using a prespecified case report form. Organisms considered clinically insignificant were excluded.

**Findings:**

We analysed data from 48 902 patients admitted to hospital between Feb 6 and June 8, 2020. The median patient age was 74 years (IQR 59–84) and 20 786 (42·6%) of 48 765 patients were female. Microbiological investigations were recorded for 8649 (17·7%) of 48 902 patients, with clinically significant COVID-19-related respiratory or bloodstream culture results recorded for 1107 patients. 762 (70·6%) of 1080 infections were secondary, occurring more than 2 days after hospital admission. *Staphylococcus aureus* and *Haemophilus influenzae* were the most common pathogens causing respiratory co-infections (diagnosed ≤2 days after admission), with Enterobacteriaceae and *S aureus* most common in secondary respiratory infections. Bloodstream infections were most frequently caused by *Escherichia coli* and *S aureus*. Among patients with available data, 13 390 (37·0%) of 36 145 had received antimicrobials in the community for this illness episode before hospital admission and 39 258 (85·2%) of 46 061 patients with inpatient antimicrobial data received one or more antimicrobials at some point during their admission (highest for patients in critical care). We identified frequent use of broad-spectrum agents and use of carbapenems rather than carbapenem-sparing alternatives.

**Interpretation:**

In patients admitted to hospital with COVID-19, microbiologically confirmed bacterial infections are rare, and more likely to be secondary infections. Gram-negative organisms and *S aureus* are the predominant pathogens. The frequency and nature of antimicrobial use are concerning, but tractable targets for stewardship interventions exist.

**Funding:**

National Institute for Health Research (NIHR), UK Medical Research Council, Wellcome Trust, UK Department for International Development, Bill & Melinda Gates Foundation, EU Platform for European Preparedness Against (Re-)emerging Epidemics, NIHR Health Protection Research Unit (HPRU) in Emerging and Zoonotic Infections at University of Liverpool, and NIHR HPRU in Respiratory Infections at Imperial College London.

## Introduction

Bacterial co-infections and secondary infections are commonly identified in severe influenza (23% in a meta-analysis)^1^ and other severe respiratory viral infections, in which they are associated with increased morbidity and mortality.^2^ National and international COVID-19 guidelines vary in their recommendations on empirical antimicrobial therapy—some recommend empirical antimicrobial therapy in severe disease,^3^, ^4^ whereas others do not.^5^ UK guidelines advise against empirical therapy when lower respiratory tract infection is thought to be due to COVID-19, without specific evidence of bacterial infection.^6^, ^7^ Living systematic reviews and meta-analyses have reported a low prevalence of confirmed bacterial co-infection (8%), but a high proportion of patients with COVID-19 received antimicrobials (pooled prevalence 75%).^8^, ^9^ The collective implication of these studies is a widespread failure of antimicrobial stewardship with the potential to worsen the global antimicrobial resistance crisis.^10^

**Research in context**

**Evidence before this study**

An understanding of co-infections, secondary infections, and patterns of antimicrobial use in patients with COVID-19 is required to inform optimal empirical antimicrobial management strategies. We searched PubMed on Dec 29, 2020, for studies published in English, using the keywords “COVID-19”, “SARS-CoV-2”, “bacterial infection”, “bacterial pneumonia”, “superinfection”, “co-infection”, “secondary infection”, and “nosocomial infection”. Systematic reviews were included in the search. A low incidence of bacterial respiratory and bloodstream infections has been consistently reported in patients admitted to hospital with COVID-19. This finding is supported by a systematic review and meta-analysis, but individual studies lack granularity in microbiological and clinical details, are retrospective, and report on small cohorts. The timing of infection (co-infection or secondary infection) is often unknown. Reporting of positive results has lacked species-level detail on cultured organisms and exclusion of clinically irrelevant pathogens. PCR-based bacterial diagnosis is associated with higher pathogen detection than culture-based methods. Antimicrobial use in inpatients appears to be high, but studies infrequently report the drug classes used.

**Added value of this study**

This is a large, multicentre, prospective cohort study reporting microbiological findings and antimicrobial use in patients admitted to hospital with COVID-19 undergoing standardised microbiological investigation of samples submitted by clinical teams (UK Standards for Microbiology Investigations). Microbiologically confirmed bacterial co-infection (≤2 days after admission) and secondary infections (>2 days after admission) were uncommon in patients admitted to hospital with COVID-19, supporting the findings of a meta-analysis of smaller studies. When they did occur, bloodstream and respiratory infections were more likely to be secondary infections and Gram-negative organisms and *Staphylococcus aureus* were key aetiological agents, adding granularity in microbiological detail. Aetiology was affected by the timing of infection onset (co-infection or secondary infection), critical care admission, and chronic pulmonary disease. We confirmed high inpatient antimicrobial usage, additionally finding evidence of regional variation, frequent use of broad-spectrum empirical therapy for lower respiratory tract infections (β-lactam–β-lactamase inhibitor combinations), and use of carbapenems rather than carbapenem-sparing alternatives.

**Implications of all the available evidence**

Bacterial co-infections and secondary infections are rare in patients admitted to hospital with COVID-19. Most infections are secondary. Gram-negative organisms and *S aureus* are important pathogens and should be considered when designing empirical antimicrobial guidelines, but emphasis should be placed on restricting empirical prescribing, especially at hospital admission. The high frequency and nature of inpatient antimicrobial use might have long-term negative consequences for antimicrobial resistance. Specific targets exist for antimicrobial stewardship and should be integrated into COVID-19 patient care pathways.

Most studies to date have been retrospective with small sample sizes, and few have systematically reported the spectrum of bacteria, timing of infection onset, or described the frequency and nature of antimicrobials used to treat them. There is an urgent need to characterise the causes of bacterial infections in patients admitted to hospital with COVID-19 to determine optimal empirical antimicrobial management strategies and identify targets for antimicrobial stewardship interventions.

In this prospective, multicentre, cohort study of patients admitted to hospital with COVID-19, we aimed to report the microbiological details of laboratory-confirmed co-infections and secondary infections identified by culture-based diagnostics, assess the effect of COVID-19-related co-infection on in-hospital mortality among patients admitted to critical care, and evaluate the nature of antimicrobial usage.

## Methods

### Study design and setting

The International Severe Acute Respiratory and Emerging Infections Consortium (ISARIC) WHO Clinical Characterisation Protocol UK (CCP-UK) study is an ongoing, prospective cohort study recruiting inpatients from 260 hospitals in England, Scotland, and Wales (National Institute for Health Research [NIHR] Clinical Research Network Central Portfolio Management System ID 14152) and conducted by the ISARIC Coronavirus Clinical Characterisation Consortium (ISARIC4C). Ethics approval was given by the South Central—Oxford C Research Ethics Committee in England (13/SC/0149), the Scotland A Research Ethics Committee (20/SS/0028), and the WHO Ethics Review Committee (RPC571 and RPC572; April, 2013). The study protocol and further details are available online.^11^ This report of microbiological findings and antimicrobial use from the study has been prepared in accordance with the STROBE statement.

### Study participants and data collection

Patients with a confirmed or clinician-defined high likelihood of SARS-CoV-2 infection were eligible for inclusion in the ISARIC WHO CCP-UK study. For this specific study, we excluded patients with a recorded negative SARS-CoV-2 test result and those without a recorded outcome at 28 days after admission (discharged alive and expected to survive, palliative discharge [not expected to survive], ongoing hospitalisation, transfer to another facility [eg, for rehabilitation], or death). Demographic, clinical, laboratory, therapeutic, and outcome data were collected using a prespecified case report form. Comorbidities were defined by a modified Charlson comorbidity index and obesity was clinician defined. Data were uploaded to a Research Electronic Data Capture Database (REDCap; Vanderbilt University, Nashville, TN USA; hosted by University of Oxford, Oxford, UK).^12^ The requirement for consent for data collection was waived in view of the public health emergency.

### Infection data processing

Microbiological data comprising organism name(s), specimen type, result status, and date of sample were recorded as a combination of dropdown fields and free text in REDCap. Results of superficial microbiology, screening swabs, and serology were excluded because of insufficient clinical information to allow interpretation. Additionally, respiratory viruses other than SARS-CoV-2 were excluded since most laboratories prioritised SARS-CoV-2 over multiplex PCR testing during the first wave of the pandemic. Except for urine samples, staphylococci, other than *Staphylococcus aureus* and *Staphylococcus lugdunensis*, were considered clinically insignificant and excluded. Unspecified organisms (including positive microscopy findings with no culture result recorded) and results recorded as mixed growth or contaminant were excluded from all sample types. *Corynebacterium* sp and *Cutibacterium* sp were excluded from blood cultures. *Candida* sp were excluded from respiratory samples.

Positive cultures from blood, sputum, or deep respiratory tract (endotracheal aspirate, bronchoalveolar lavage, and pleural fluid) samples were considered possible COVID-19-related infections, while growth from other sample types was considered unrelated. Clinically significant positive results from samples collected within 2 days of admission were categorised as co-infections, and those collected more than 2 days after admission as secondary infections, synonymous with hospital onset or hospital-acquired infection.^13^ A patient could be recorded as having multiple infections, based on different sample types and different timepoints, during their admission.

Antimicrobials prescribed over the course of admission were entered as free text and mapped to drug names found in the UK National Health Service Technology Reference Data Update Distribution system.

### Statistical analysis

Categorical variables are summarised as frequencies and percentages. Continuous variables are presented as means and SDs or medians and IQRs, depending on data distribution. Analysis of antimicrobial co-occurrence was done using the Jaccard similarity index and represented visually as heatmaps. p≤0·05 was considered to indicate a statistically significant difference. All statistical analyses were done using R version 3.6.3 using the *Tidyverse, finalfit, stringdist*, and *fuzzyjoin* packages.

### Role of the funding source

The funder of the study had no role in study design, data collection, data analysis, data interpretation, or writing of the report.

## Results

**Figure 1:**
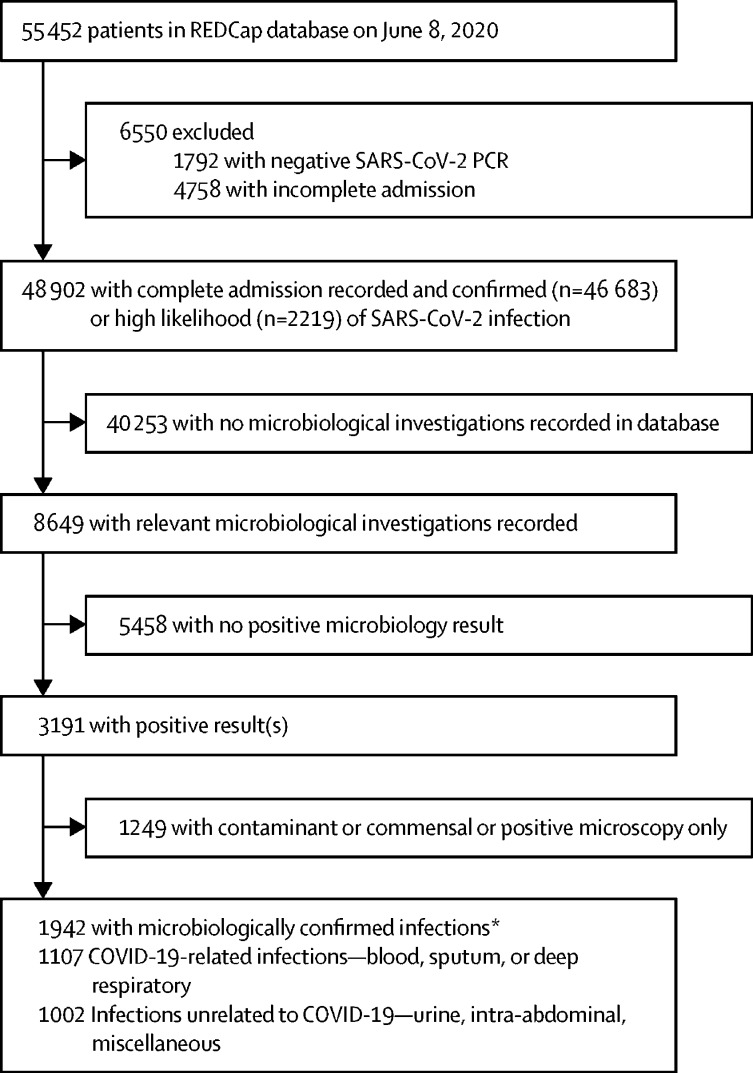
Study flowchart A complete admission was considered an outcome recorded at 28 days or earlier, and could either be a final outcome (eg, death or discharged alive) or documentation the patient remained in hospital. Relevant microbiological investigations were blood cultures, sputum, deep respiratory (endotracheal aspirates, bronchoalveolar lavage, and pleural fluid), urine, abdominopelvic, or pus samples from abscesses. *Some patients had both a COVID-19 related and unrelated infection.

We analysed data from 48 902 patients admitted to hospital between Feb 6 and June 8, 2020 (figure 1). The median patient age was 74 years (IQR 59–84) and 20 786 (42·6%) of 48 765 patients were female (table). The most common comorbidities were chronic cardiac disease and chronic pulmonary disease excluding asthma (table). 13 390 (37·0%) of 36 145 patients had received antimicrobials in the community for this illness episode before hospital admission. Vital signs and laboratory results on admission are shown in the appendix (p 1). 31 422 (69·2%) of 45 420 patients received supplemental oxygen during their hospital stay, 6755 (14·9%) of 45 420 patients received non-invasive ventilation, and 4241 (9·3%) of 45 607 patients received invasive mechanical ventilation (table). 7090 (14·5%) of 48 902 patients required admission to critical care (high dependency or intensive care unit). 15 392 (31·5%) of 48 902 inpatients died in hospital.

**Table:** Baseline characteristics, management, and outcomes of a cohort of 48 902 patients with COVID-19

		**n**	**Patients with COVID-19**
**Demographics**			
Sex		48 765	..
	Male	..	27 979 (57·4%)
	Female	..	20 786 (42·6%)
Age, years		48 902	74·0 (59·0–84·0)
**Comorbidities**			
Chronic cardiac disease		45 781	14 775 (32·3%)
Hypertension		22 006	10 645 (48·4%)
Chronic pulmonary disease		45 909	13 169 (28·7%)
Immunocompromise		25 742	7548 (29·3%)
Asthma		45 470	6309 (13·9%)
Chronic kidney disease		45 451	8092 (17·8%)
Chronic liver disease (moderate or severe)		45 001	867 (1·9%)
Chronic liver disease (mild)		44 880	683 (1·5%)
Chronic neurological disease		45 187	5747 (12·7%)
Current malignancy		45 067	4730 (10·5%)
Chronic haematological disease		45 019	1984 (4·4%)
HIV/AIDS		44 510	184 (0·4%)
Obesity*		41 058	4877 (11·9%)
Diabetes		46 180	13 581 (29·4%)
Rheumatological disease		44 946	5065 (11·3%)
Dementia		45 310	7534 (16·6%)
Smoking		31 125	..
	Never	..	17 953 (57·7%)
	Former	..	10 825 (34·8%)
	Current	..	2347 (7·5%)
**Pre-admission**			
Symptom duration before admission, days		..	4·0 (1·0–8·0)
Receipt of antimicrobials before admission		36 145	13 390 (37·0%)
**Maximum level of respiratory support**			
Non-invasive ventilation		45 420	6755 (14·9%)
Invasive mechanical ventilation		45 607	4241 (9·3%)
**Other organ support**			
Renal replacement therapy		44 803	1508 (3·4%)
Vasopressors		44 672	2987 (6·7%)
**Medical management**			
Antibacterial		46 061	39 258 (85·2%)
Antifungal		48 902	309 (0·6%)
Corticosteroids		48 902	4850 (9·9%)
**Outcomes**			
Outcome at ≥28 days		48 902	..
	Discharged alive and expected to survive	..	28 819 (58·9%)
	Ongoing hospitalisation	..	1186 (2·4%)
	Transfer to other facility	..	2623 (5·4%)
	Inpatient death	..	15 392 (31·5%)
	Palliative discharge	..	869 (1·8)
	Unknown or awaited	..	13 (<0·1%)
Length of stay, days†		..	9·0 (4·0–17·0)

Data are n (%) or median (IQR).

* Clinician defined.

† Excludes patients remaining in hospital after 28 days.

Microbiological investigations were recorded for 8649 (17·7%) of 48 902 patients (figure 1; appendix p 1). Characteristics of this subgroup of patients are presented in the appendix (p 2). After exclusion of negative and non-significant results, we observed 2109 clinically significant results for 1942 patients. Patients admitted to critical care were more likely to have a documented blood or respiratory culture than ward-level patients (2163 [30·5%] of 7090 patients *vs* 5430 [13·0%] of 41 812 patients; p<0·0001). Positivity rates of recorded cultures were high—602 (42·1%) of 1429 cultures for sputum, 207 (51·5%) of 402 cultures for deep respiratory samples, and 500 (8·1%) of 6157 cultures for blood. We concluded that there was a bias towards recording positive results; therefore, we elected not to estimate the incidence of any infection within the cohort, restricting the reporting of proportions to identified organisms within positive samples.

We identified 1107 patients with COVID-19-related infections (positive blood, sputum, or deep respiratory cultures) and 1002 patients with unrelated infections (urine, abdominopelvic, or pus or abscess cultures). For microbiologically confirmed COVID-19-related infections where sample timing was known, 762 (70·6%) of 1080 infections were secondary, occurring more than 2 days after hospital admission. Among patients admitted to critical care, we found no association between patients identified to have a respiratory or bloodstream infection and inpatient mortality on univariable analysis (unadjusted odds ratio 1·02, 95% CI 0·86–1·22; p=0·81).

**Figure 2:**
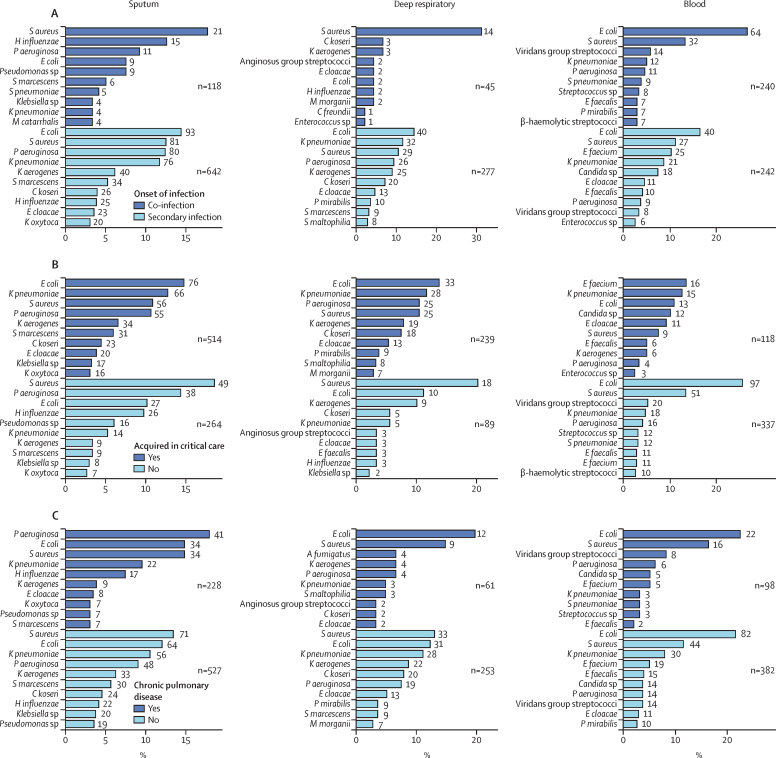

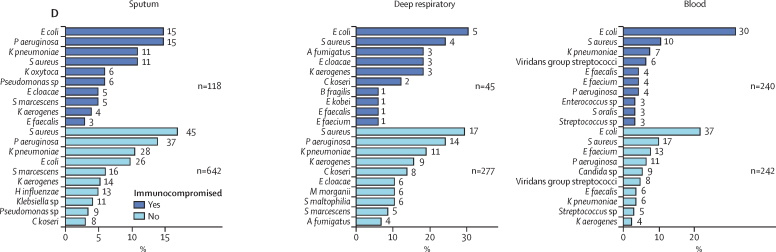
Cause of microbiologically confirmed respiratory and bloodstream infections The ten most identified pathogens from sputum, deep respiratory, and blood cultures, as a percentage of all positive samples of that type. The number at the end of each bar represents the total number of positive samples for the pathogen. Numbers annotated on the plots indicate the total number of organisms for each subgroup. Deep respiratory samples are endotracheal aspirates, bronchoalveolar lavage, and pleural fluid. Pathogen identification is stratified into sample obtained <2 days (co-infection) or >2 days (secondary infection) from admission to hospital (A); sample obtained <1 day or >1 day after admission to critical care (B); presence or absence of chronic pulmonary disease (C); and presence or absence of immunocompromise (D). *S aureus*=*Staphylococcus aureus. H influenzae*=*Haemophilus influenzae. P aeruginosa*=*Pseudomonas aeruginosa. E coli*=*Escherichia coli. S marcescens*=*Serratia marcescens. S pneumoniae*=*Streptococcus pneumoniae. K pneumoniae*=*Klebsiella pneumoniae. M catarrhalis*=*Moraxella catarrhalis. K aerogenes*=*Klebsiella aerogenes. C koseri*=*Citrobacter koseri. E cloacae*=*Enterobacter cloacae. K oxytoca*=*Klebsiella oxytoca. M morganii*=*Morganella morganii. C freundii*=*Citrobacter freundii. P mirabilis*=*Proteus mirabilis. S maltophilia*=*Stenotrophomonas maltophilia. E faecalis*=*Enterococcus faecalis. E faecium*=*Enterococcus faecium. A fumigatus*=*Aspergillus fumigatus. B fragilis*=*Bacteroides fragilis. E kobei*=*Enterobacter kobei. S oralis*=*Streptococcus oralis*.

607 respiratory infections were recorded. Most culture-positive respiratory samples represented secondary infections (919 [84·9%] of 1082 sputum and deep respiratory cultures; figure 2A). In lower respiratory tract co-infections (within 2 days of admission), *S aureus* (21 [17·8%] of 118 organisms), *Haemophilus influenzae* (15 [12·7%] organisms), and *Pseudomonas aeruginosa* (11 [9·3%] organisms) were most frequently identified in sputum (figure 2). *Streptococcus pneumoniae* was infrequently cultured (five [4·2%] of 118 organisms). Among deep respiratory samples identifying co-infection, *S aureus* was the predominant organism (14 [31·1%] of 45 organisms). *S aureus* was also a common cause of secondary lower respiratory tract infections (sputum 81 [12·6%] of 642 organisms; deep respiratory 29 [10·5%] of 277 organisms), but most organisms were Gram-negative, including *Escherichia coli* (sputum 93 [14·5%] of 642 organisms; deep respiratory 40 [14·4%] of 277 organisms), *P aeruginosa* (sputum 80 [12·5%] of 642 organisms; deep respiratory 26 [9·4%] of 277 organisms), *Klebsiella pneumoniae* (sputum 76 [11·8%] of 642 ogranisms; deep respiratory 32 [11·6%] of 277 organisms), *Klebsiella aerogenes* (sputum 40 [6·2%] of 642 organisms; deep respiratory 25 [9·0%] of 277 organisms), and *Citrobacter koseri* (sputum 26 [4·0%] of 642 organisms; deep respiratory 20 [7·2%] of 277 organisms).

When stratified by critical care admission status, 174 (72·8%) of 239 organisms were identified from deep respiratory samples obtained more than 1 day after critical care admission (figure 2B). Deep respiratory samples included bronchoalveolar lavage, endotracheal aspirate, and other distal lung samples; the microbiological results from each of these sample types were similar (appendix pp 3, 5). In the critical care setting, *E coli* was the most common cause of lower respiratory tract infections (sputum 76 [14·8%] of 514 organisms; deep respiratory 33 [13·8%] of 239 organisms), followed by *K pneumoniae* (sputum 66 [12·8%] of 514 organisms; deep respiratory 28 [11·7%] of 239 organisms), *S aureus* (sputum 56 [10·9%] of 514 organisms; deep respiratory 25 [10·5%] of 239 organisms), and *P aeruginosa* (sputum 55 [10·7%] of 514 organisms; deep respiratory 25 [10·5%] of 239 organisms). Several opportunistic organisms—such as *C koseri, Stenotrophomonas maltophilia*, and *Morganella morganii*—were cultured from respiratory samples obtained in critical care. Among non-critical care patients, *S aureus* was the most common organism in sputum (49 [18·6%] of 264 organisms) and deep respiratory samples (18 [20·2%] of 89 organisms), followed by *P aeruginosa* (sputum 38 [14·4%] of 264 organisms; deep respiratory none of 89 organisms), *E coli* (sputum 27 [10·2%] of 264 organisms; deep respiratory ten [11·2%] of 89 organisms), and *H influenzae* (sputum 26 [9·8%] of 264 organisms; deep respiratory three [3·4%] of 89 organisms).

Sputum microbiology differed between patients with and without chronic lung disease (figure 2C). Although *S aureus* (71 [13·5%] of 527 organisms), *E coli* (64 [12·1%] organisms), and *K pneumoniae* (56 [10·6%] organisms) were the predominant organisms detected in patients without chronic respiratory disease, *P aeruginosa* (41 [18·0%] of 228 organisms), *E coli* (34 [14·9%] organisms), and *S aureus* (34 [14·9%] organisms) were the most frequently isolated in those with chronic lung disease. *Aspergillus fumigatus* was also cultured from deep respiratory samples in four patients with chronic lung disease, three of whom were immunocompromised (clinician-defined or in receipt of immunomodulatory therapy before admission to hospital). We observed no other major differences in lower respiratory tract microbiology stratified by immune status (figure 2D).

With regard to blood cultures, 500 clinically significant results were recorded (appendix p 1). *E coli* (104 [20·8%] of 500 organisms) was the most frequently identified pathogen, followed by *S aureus* (59 [11·8%]; figure 2) and *K pneumoniae* (33 [6·6%]; figure 2). Pneumococcal bacteraemia was uncommon (nine [1·8%] of 500 organisms). In bacteraemia occurring more than 1 day after admission to critical care, *Enterococcus faecium* (16 organisms) and *K pneumoniae* (15 organisms) were the most common pathogens. Candidaemia (12 organisms) was also more common. We observed no major differences when patients were stratified by immune status but viridans group streptococci and *P aeruginosa* were over-represented in patients with chronic lung disease. To determine the source of bacteraemia, we identified patients with the same organism isolated from another sample type. Generally, we found little overlap between blood and other cultures, with more overlap observed for sputum and deep respiratory samples compared with other samples (appendix p 4). *S aureus* was cultured from a sputum sample in nine (15·0%) of 60 patients with *S aureus* bacteraemia. Despite the frequency of *E coli* bacteraemia, *E coli* bacteriuria in the same patients was infrequently identified (appendix p 4).

**Figure 3:**
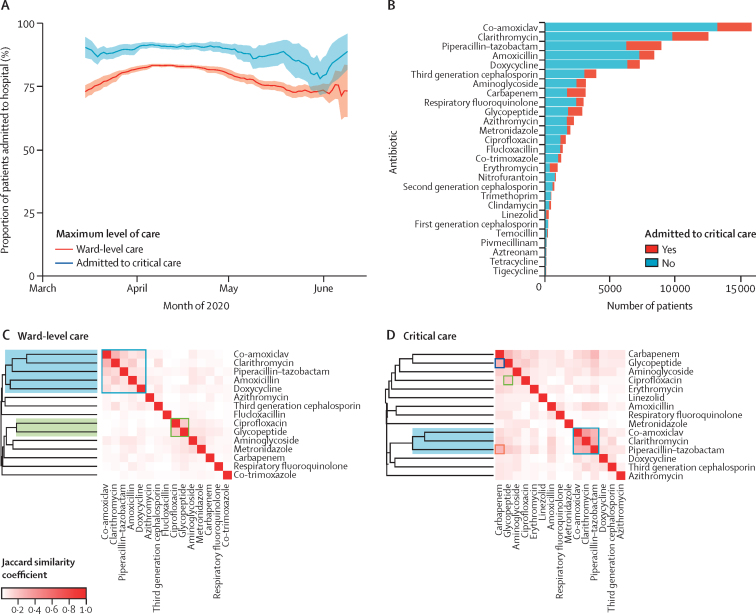
Frequency and nature of antimicrobial use (A) The proportion of inpatients receiving antimicrobials between March and June, 2020, stratified by level of care. Data show rolling mean over a window of 14 days. Dotted lines represent 95% CIs. (B) Specific antimicrobials used, stratified by level of care or critical care. Co-use of specific antimicrobials in patients receiving ward-level care (C) and patients admitted to critical care (D), highlighting antimicrobials used for lower respiratory tract infection (blue box and shading), a signature of prescribing in response to penicillin allergy (green box and shading), carbapenem and glycopeptide usage in critical care (D only; blue box) and piperacillin–tazobactam and carbapenem in critical care (D only; orange box). The greater the intensity of red shading, the greater the correlation of antimicrobial use measured by Jaccard distance. Dendrograms show the result of hierarchical clustering. Data available for 46 061 patients.

**Figure 4:**
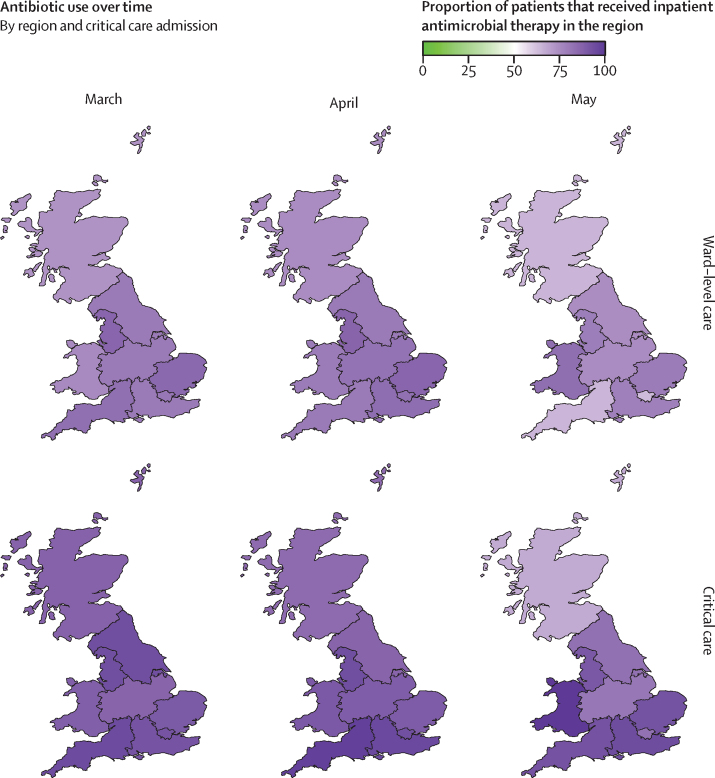
Geographical variation in antimicrobial use over time Maps are divided into Scotland, Wales, and regions of England (northeast and Yorkshire, northwest, midlands, east, southeast, southwest, and London). Purple shading of regions represents the percentage of patients who received antimicrobial therapy during their hospital admission, stratified by month of admission (March, April, and May) and by level of care (ward-level or critical care). Data available for 46 061 patients.

39 258 (85·2%) of 46 061 patients with inpatient antimicrobial data received one or more antimicrobials at some point during their admission. Antimicrobial use was highest in March and April, 2020, both among patients admitted and not admitted to critical care (figure 3A). This proportion reduced over the course of May, 2020. We found substantial regional differences in prescribing, in both the proportion of patients given antimicrobials (figure 4; appendix pp 6–7) and the type of antimicrobial prescribed (appendix pp 6–7). Across all regions, a higher proportion of patients admitted to critical care received antimicrobials compared with ward-level patients. The proportion of patients receiving antimicrobials fell or remained static across most regions; the largest decline was observed in Scotland in critical care (150 [87·2%] of 172 patients in March, 2020, to 12 [66·7%] of 18 patients in May, 2020), and in the southwest of England in ward-level care (678 [82·7%] of 820 patients in March, 2020, to 200 [63·9%] of 313 patients in May, 2020). Amoxicillin was more commonly prescribed in Scotland and Wales compared with England, whereas co-amoxiclav prescription was higher in England than Scotland and Wales, particularly among ward-level patients (appendix pp 6–7). Piperacillin-tazobactam prescription among ward-level patients was higher in Wales and certain English regions, including the northwest, southwest, east, and northeast and Yorkshire, compared with other regions.

β-lactam–β-lactamase inhibitor combinations were among the top three most prescribed antimicrobials, accounting for 30·0% of total prescriptions (co-amoxiclav 19·2%, piperacillin–tazobactam 10·8%; figure 3B). Narrower-spectrum antimicrobials used for the treatment of lower respiratory tract infections accounted for fewer prescriptions (amoxicillin 10·2% and doxycycline 8·9%). Consistent with lower respiratory tract infections being a major indication for antimicrobial use in this cohort, hierarchical clustering identified a cluster of clarithromycin, β-lactam–β-lactamase inhibitors, amoxicillin, and doxycycline in patients receiving ward-level care (figure 3C). The strongest correlation for co-receipt was between co-amoxiclav and clarithromycin (Jaccard similarity coefficient 0·29). We observed a similar cluster in patients admitted to critical care, but this did not include amoxicillin or doxycycline (figure 3D). A cluster of the combination of a glycopeptide with ciprofloxacin was observed in patients receiving both ward-level (figure 3C) and critical care (figure 3D). Among patients admitted to critical care, a high proportion received piperacillin–tazobactam, carbapenems, and glycopeptides, with evidence of co-prescription of carbapenem and glycopeptide (figure 3D), as well as piperacillin–tazobactam and carbapenem (figure 3D). Carbapenems accounted for 3·8% of all prescriptions, whereas carbapenem-sparing Gram-negative active alternatives^14^ were less frequently prescribed (co-trimoxazole 1·5%, temocillin 0·3%, and aztreonam 0·2%). Almost half of prescriptions involved WHO AWaRe watch antimicrobials (appendix p 8).

## Discussion

In this multicentre, prospective cohort of 48 902 patients admitted to hospital with COVID-19, microbiologically confirmed infections were infrequent (1107 patients). Co-infection at hospital admission was rare and when infections were identified, most were secondary. Gram-negative organisms and *S aureus* were the most frequently recovered pathogens from respiratory and blood cultures. These findings have implications for empirical therapy, indicating a need to treat Gram-negative bacteria and *S aureus* empirically until culture results are available. Most patients received antimicrobials, which were commonly broad spectrum. We found geographical heterogeneity in antimicrobial usage, reflecting variations in regional and local practices.

Our findings support and add granularity to those of a living systematic review and meta-analysis of 38 studies that examined bacterial infections in patients with COVID-19.^8^ Langford and colleagues reported a pooled prevalence of confirmed bacterial infections of 8·0% (95% CI 6·1–9·9), with a higher prevalence of secondary infections (16·0%, 12·4–19·6) than co-infections (4·9%, 2·6–7·1).^8^ Although this meta-analysis included more than 6000 patients, most of the included studies were retrospective and small (13 studies had <50 patients). Moreover, the timing of infection, microbiological details, and antimicrobial use were infrequently reported. By contrast, we included microbiological data derived from standardised laboratory investigations^15^ from a cohort of nearly 50 000 patients. We restricted analyses to clinically significant results, correlated with the timing of sample collection, and described antimicrobial use over time and by geographical region. The multicentre design of our study improves the generalisability of our findings.

The pattern of infection and associated pathogens in our cohort of patients with COVID-19 differs from that reported in influenza. *S aureus* and *H influenzae* were the most common (but infrequent) co-infecting respiratory pathogens*,* and bacteria identified in secondary infections and critical care were predominantly Gram-negative organisms, including Enterobacteriaceae and *P aeruginosa*, in addition to *S aureus* (ie, organisms typically identified in hospital-acquired or ventilator-acquired pneumonia). By contrast, bacterial co-infections are much more common among patients with severe influenza,^1^ and are predominantly caused by *S pneumoniae* or *S aureus*.^1^ Other studies of patients with COVID-19 have identified *S aureus* as a frequent cause of infection, including bacteraemia. We were unable to definitively determine the sources of bacteraemia in our study. However, in a case series of *S aureus* bacteraemia in 41 patients with COVID-19, most cases of bacteraemia were of unknown origin (69%), followed by pneumonia (19%), then vascular causes, including vascular access devices (7·1%).^16^ Exclusion of coagulase-negative staphylococci from our analysis might have led to the under-ascertainment of central line-associated bloodstream infections.

Despite little evidence of bacterial infections in our cohort, a high proportion of patients received antimicrobials. This finding is consistent with a meta-analysis that reported a pooled prevalence of antimicrobial usage of around 75% in patients with COVID-19.^9^ Elevated C-reactive protein and radiological pulmonary infiltrates are often used to differentiate bacterial from viral causes in community-acquired pneumonia. However, both of these findings are commonly present in patients with COVID-19.^17^, ^18^ In a Scottish survey, C-reactive protein levels of at least 100 mg/L and chronic obstructive pulmonary disease or chronic lung disease were positively associated with antimicrobial prescribing.^19^ Combined with high morbidity and mortality, the absence of effective therapeutic options for COVID-19 during the study period, and clinical uncertainty during acute illness, high rates of antimicrobial prescription are unsurprising. Nevertheless, the infrequency of confirmed co-infection supports restrictive empirical antimicrobial usage. Antimicrobials should be restricted to individuals with atypical features of COVID-19, such as purulent sputum or lobar pneumonia, or evidence of distinct non-respiratory co-infection. Blood and sputum cultures before empirical antimicrobial treatment and incorporating trends in inflammatory markers into decision making could also support judicious antimicrobial use. The absence of an elevated white cell count at baseline and antimicrobial-associated C-reactive protein reduction can exclude co-infection in around 50% of patients with COVID-19.^20^ Procalcitonin might be an additional decision-making adjunct, using a threshold of 0·25 ng/mL or less to identify patients with a reduced likelihood of bacterial infection.^21^ Regular review of drug charts with discontinuation of antimicrobials if co-infection is deemed unlikely, supported by negative microbiological investigations, are also key to minimise unnecessary antimicrobial exposure. When antimicrobials are required, the choice of antimicrobial should be tailored to likely pathogens and local resistance patterns, with treatment duration limited to 5 days if lower respiratory tract infection is suspected.^6^, ^7^, ^22^ Co-prescription of glycopeptides and ciprofloxacin was identified, which we postulate is a signature of prescribing in response to penicillin allergy labels. Many patients can be de-labelled without the need for penicillin challenges, associated with reduced broad-spectrum antimicrobial use, and this strategy should be encouraged in patients admitted to hospital with COVID-19 in advance of a potential need for antimicrobials.^23^ Furthermore, we identified frequent use of broad-spectrum empirical therapy (β-lactam–β-lactamase inhibitors) for lower respiratory tract infections, empirical escalation from piperacillin–tazobactam to carbapenems in critical care, and preferential use of carbapenems over carbapenem-sparing alternatives. These findings all represent tractable targets for antimicrobial stewardship.

In contrast to other studies,^24^ co-infection and secondary infection were not associated with inpatient mortality among patients admitted to critical care in our study. Evaluation of the association between co-infection and outcome using observational data is complex. First, a greater proportion of deaths within the study occurred early during the hospital stay; therefore, these patients had less time to undergo microbiological investigations or to acquire a secondary infection. Conversely, any observed association could be driven by prolonged treatment in hospital because of more severe disease or slower recovery—co-infection or secondary infection might simply be a marker of this. Additionally, more thorough investigations might be done in patients who appear likely to have a favourable prognosis with adequate treatment, and such patients might be younger with fewer comorbidities.

Our study has several limitations. Retrospectively assigning clinical significance to culture results can be challenging and is further restricted in this study, as clinical findings that would contribute to diagnosis of bacterial infection were not collected at the time of microbiological sampling. The absence of clinical correlation limits interpretation of the microbiological findings—eg, isolation of *E coli* from sputum could represent true infection but alternative explanations include airway colonisation or altered (post-antimicrobial) flora, particularly in patients with chronic lung disease. We were unable to differentiate colonisation from new infection for patients with chronic lung disease, as previous microbiology results were not available.

Microbiological diagnosis of bacterial or fungal co-infection is itself challenging, especially in the context of COVID-19. Fewer diagnostic procedures might have been done during the pandemic because of clinical pressure and concerns regarding health-care worker safety (particularly from aerosol generating procedures, such as bronchoalveolar lavage), and inter-site variation in microbiological sampling is likely.^25^ Less than 20% of our cohort had microbiological investigations recorded, which is lower than reported for community-acquired pneumonia in a recent UK-wide audit (sputum and blood cultures were done in 23·6% of patients and 58·6% of patients, respectively).^26^ Low rates of microbiological sampling in patients with COVID-19 have been reported elsewhere.^27^ Under-ascertainment of true bacterial infection might also be due to antimicrobial receipt before sampling, as well as low sensitivity of culture-based diagnostics. We excluded serological investigations for fungi or atypical bacteria, and urinary antigen tests for pneumococci and *Legionella* sp, as these were inconsistently recorded and the galactomannan assay cross-reacts with β-lactams.^28^ We also lacked the data required to address the recently proposed case definition for COVID-19-associated pulmonary aspergillosis.^29^ Furthermore, we were unable to characterise respiratory viral or *Mycoplasma pneumoniae* co-infections, as most UK laboratories discontinued routine multiplex respiratory virus PCR testing during the study period. Therefore, we might have missed fungal or atypical bacterial co-infection identified by serum or urinary antigen tests alone, and other respiratory pathogen infections usually diagnosed by PCR.

We suspect the preferential recording of positive microbiology results based on the high culture-positivity rates; this limits our ability to determine an accurate denominator (ie, total number of microbiological investigations done); thus we have refrained from prevalence estimates. This factor might partly explain the apparently low rates of microbiological investigations we observed. The timing of initiation and duration of antimicrobial therapy, and data on antibiograms of identified organisms, were not recorded. Hence, we were unable to link infections with prescribed antimicrobials or resistance patterns—eg, the proportion of *S aureus* that are meticillin resistant. Nonetheless, this proportion is likely to be low due to low meticillin-resistant *S aureus* infection rates in the UK.^30^

In conclusion, microbiologically confirmed bacterial or fungal infections were rare in patients admitted to hospital with COVID-19 during the first wave of the pandemic in the UK. When such infections occurred, they were mostly secondary and were caused by Gram-negative organisms and *S aureus*. The epidemiology of bacterial and fungal infections might change as immunomodulatory therapy for patients with COVID-19 progresses. Short-term systemic corticosteroid use in outpatients has been associated with a small increased risk of sepsis, and IL-6 inhibition outside clinical trials is associated with increased risk of bacterial infection.^31^, ^32^ Prospective studies with standardised and comprehensive microbiological sampling before antimicrobial treatment are needed to characterise co-infections and secondary infections in patients with COVID-19. Tractable targets for antimicrobial stewardship interventions exist and should be prioritised.

## Data sharing

Access to all data and samples collected by ISARIC4C are controlled by an Independent Data and Materials Access Committee comprising representatives of research funders, academia, clinical medicine, public health, and industry. The application process for access to the data is available on the ISARIC4C website.

## Declaration of interests

All authors declare support from the NIHR, the Medical Research Council (MRC), the NIHR Health Protection Research Unit (HPRU) in Emerging and Zoonotic Infections at University of Liverpool, the NIHR HPRU in Respiratory Infections at Imperial College London, the NIHR Biomedical Research Centre (BRC) at Imperial College London, and the NIHR Clinical Research Network, for the submitted work. ABD reports grants from the UK Department of Health and Social Care (DHSC), during the conduct of the study, and grants from Wellcome Trust, outside the submitted work. PJMO reports personal fees from consultancies (GlaxoSmithKline, Janssen, Bavarian Nordic, Pfizer, and Cepheid) and from the European Respiratory Society, grants from MRC, MRC Global Challenge Research Fund, the EU, NIHR BRC, MRC–GlaxoSmithKline, Wellcome Trust, NIHR (HPRU in Respiratory Infection), and is an NIHR senior investigator outside the submitted work. PJMO's role as President of the British Society for Immunology was unpaid but travel and accommodation at some meetings was provided by the Society. JKB reports grants from MRC. MGS reports grants from DHSC, NIHR UK, MRC, HPRU in Emerging and Zoonotic Infections, and University of Liverpool, during the conduct of the study, and is chair of the scientific advisory board and a minority share holder at Integrum Scientific, outside the submitted work.

## References

[1] Klein EY, Monteforte B, Gupta A (2016). The frequency of influenza and bacterial coinfection: a systematic review and meta-analysis. Influenza Other Respir Viruses.

[2] Joseph C, Togawa Y, Shindo N (2013). Bacterial and viral infections associated with influenza. Influenza Other Respir Viruses.

[3] WHO (2021). Clinical management of COVID-19: living guidance. Jan 25, 2021.

[4] Alhazzani W, Møller MH, Arabi YM (2020). Surviving sepsis campaign: guidelines on the management of critically ill adults with coronavirus disease 2019 (COVID-19). Crit Care Med.

[5] National Institutes of Health (2020). Coronavirus Disease 2019 (COVID-19) Treatment Guidelines. https://www.covid19treatmentguidelines.nih.gov/.

[6] National Institute for Health and Care Excellence (May 1, 2020). COVID-19 rapid guideline: antibiotics for pneumonia in adults in hospital. https://www.nice.org.uk/guidance/ng173/resources/prescribing-tables-to-guide-decision-making-about-antibiotic-choice-pdf-8719038253.

[7] Scottish Antimicrobial Prescribing Group (May 12, 2020). Advice to antimicrobial management teams (AMTs) on antimicrobial prescribing in suspected lower respiratory tract infections in the context of the COVID-19 pandemic. https://www.sapg.scot/media/5175/20200512-sapg-advice-on-antimicrobial-management-in-respiratory-infection-covid-19-may-2020-v1.pdf.

[8] Langford BJ, So M, Raybardhan S (2020). Bacterial co-infection and secondary infection in patients with COVID-19: a living rapid review and meta-analysis. Clin Microbiol Infect.

[9] Langford BJ, So M, Raybardhan S (2021). Antibiotic prescribing in patients with COVID-19: rapid review and meta-analysis. Clin Microbiol Infect.

[10] Lynch C, Mahida N, Gray J (2020). Antimicrobial stewardship: a COVID casualty?. J Hosp Infect.

[11] Docherty AB, Harrison EM, Green CA (2020). Features of 20 133 UK patients in hospital with COVID-19 using the ISARIC WHO Clinical Characterisation Protocol: prospective observational cohort study. BMJ.

[12] Harris PA, Taylor R, Minor BL (2019). The REDCap consortium: building an international community of software platform partners. J Biomed Inform.

[13] Cardoso T, Almeida M, Friedman ND (2014). Classification of healthcare-associated infection: a systematic review 10 years after the first proposal. BMC Med.

[14] Russell CD, Laurenson IF, Evans MH, Mackintosh CL (2019). Tractable targets for meropenem-sparing antimicrobial stewardship interventions. JAC Antimicrob Resist.

[15] Public Health England (August 1, 2014). Standards for microbiology investigations (UK SMI). https://www.gov.uk/government/collections/standards-for-microbiology-investigations-smi.

[16] Cusumano JA, Dupper AC, Malik Y (2020). *Staphylococcus aureus* bacteremia in patients infected with COVID-19: a case series. Open Forum Infect Dis.

[17] Luo X, Zhou W, Yan X (2020). Prognostic value of C-reactive protein in patients with coronavirus 2019. Clin Infect Dis.

[18] Zhou F, Yu T, Du R (2020). Clinical course and risk factors for mortality of adult inpatients with COVID-19 in Wuhan, China: a retrospective cohort study. Lancet.

[19] Seaton RA, Gibbons CL, Cooper L (2020). Survey of antibiotic and antifungal prescribing in patients with suspected and confirmed COVID-19 in Scottish hospitals. J Infect.

[20] Mason CY, Kanitkar T, Richardson CJ (2021). Exclusion of bacterial co-infection in COVID-19 using baseline inflammatory markers and their response to antibiotics. J Antimicrob Chemother.

[21] Williams EJ, Mair L, de Silva TI (2021). Evaluation of procalcitonin as a contribution to antimicrobial stewardship in SARS-CoV-2 infection: a retrospective cohort study. J Hosp Infect.

[22] Sieswerda E, de Boer MGJ, Bonten MMJ (2021). Recommendations for antibacterial therapy in adults with COVID-19—an evidence based guideline. Clin Microbiol Infect.

[23] Chua KYL, Vogrin S, Bury S (2020). The penicillin allergy delabeling program: a multicenter whole-of-hospital health services intervention and comparative effectiveness study. Clin Infect Dis.

[24] Neto AGM, Lo KB, Wattoo A (2021). Bacterial infections and patterns of antibiotic use in patients with COVID-19. J Med Virol.

[25] Lentz RJ, Colt H (2020). Summarizing societal guidelines regarding bronchoscopy during the COVID-19 pandemic. Respirology.

[26] British Thoracic Society (2019). National Audit Report: Adult Community Acquired Pneumonia Audit 2018/19. https://www.brit-thoracic.org.uk/quality-improvement/clinical-audit/national-adult-community-acquired-pneumonia-audit-201819/.

[27] Vaughn VM, Gandhi T, Petty LA (2020). Empiric antibacterial therapy and community-onset bacterial co-infection in patients hospitalized with COVID-19: a multi-hospital cohort study. Clin Infect Dis.

[28] Aubry A, Porcher R, Bottero J (2006). Occurrence and kinetics of false-positive *Aspergillus* galactomannan test results following treatment with beta-lactam antibiotics in patients with hematological disorders. J Clin Microbiol.

[29] Koehler P, Bassetti M, Chakrabarti A (2020). Defining and managing COVID-19-associated pulmonary aspergillosis: the 2020 ECMM/ISHAM consensus criteria for research and clinical guidance. Lancet Infect Dis.

[30] Public Health England (Dec 3, 2020). MRSA bacteraemia: annual data. https://www.gov.uk/government/statistics/mrsa-bacteraemia-annual-data.

[31] Lang VR, Englbrecht M, Rech J (2012). Risk of infections in rheumatoid arthritis patients treated with tocilizumab. Rheumatology (Oxford).

[32] Waljee AK, Rogers MA, Lin P (2017). Short term use of oral corticosteroids and related harms among adults in the United States: population based cohort study. BMJ.

