# Supplementary material for: Co-infections, secondary infections, and antimicrobial use in patients hospitalised with COVID-19 during the first pandemic wave from the ISARIC WHO CCP-UK study: a multicentre, prospective cohort study
Source: Lancet Microbe. 2021 Aug;2(8):e354–65. doi: 10.1016/S2666-5247(21)00090-2 (PMC8172149; doi:10.1016/S2666-5247(21)00090-2)
Supplement: Supplementary appendix [file mmc1.pdf]

THE LANCET
Microbe

Supplementary appendix

This appendix formed part of the original submission and has been peer reviewed. We post it as supplied by the authors.

Supplement to: Russell CD, Fairfield CJ, Drake TM, et al. Co-infections, secondary infections, and antimicrobial use in patients hospitalised with COVID-19 during the first pandemic wave from the ISARIC WHO CCP-UK study: a multicentre, prospective cohort study. *Lancet Microbe* 2021; published online June 2. https://doi.org/10.1016/S2666-5247(21)00090-2.

Supplementary Materials

Supplementary Table 1: Vital signs, laboratory results and thoracic radiology

Variable	Value
**Vital signs**[1]	
Temperature, °C, mean (SD)	37·4 (1·1)
Respiratory rate, breaths/min, median (IQR)	21·0 (18·0–26·0)
Systolic blood pressure, mmHg, mean (SD)	131·2 (25·4)
Heart rate, beats/min, mean (SD)	92·4 (21·9)
Oxygen saturation, %, median (IQR)	95·0 (92·0–97·0)
**Laboratory results**[1]	
Total white cell count, x10^9/L, median (IQR)	7·3 (5·3–10·3)
Neutrophil count, x10^9/L, median (IQR)	5·6 (3·8–8·4)
Lymphocyte count, x10^9/L, median (IQR)	0·9 (0·6–1·3)
Platelet count, x10^9/L, mean (SD)	236·4 (112·4)
C-reactive protein, mg/L, median (IQR)	83·0 (35·0–157·0)
Procalcitonin, ng/mL, median (IQR)	0·3 (0·1–1·1)
**Thoracic radiology**	
Thoracic imaging performed on admission[2] (n=47303)	34355 (72·6)
Infiltrates present on admission (n=31947)	11831 (37·0)

Numbers are N (%) unless otherwise stated.
[1]Recorded on admission.
[2]X-ray or CT.

Supplementary Table 2: Results of microbiological investigations

Sample type	N (%)
**Blood cultures**[1] (n=6157 recorded)	
Negative/contaminant	5461 (88·7)
Positive microscopy only	196 (3·2)
**Significant positive**[5]	**500 (8·1)**
**Sputum** (n=1429 recorded)	
Negative/contaminant	781 (54·7)
Positive microscopy only	46 (3·2)
**Significant positive**[5]	**602 (42·1)**
**Deep respiratory**[2] (n=402 recorded)	
Negative/contaminant	188 (46·8)
Positive microscopy only	7 (1·7)
**Significant positive**[5]	**207 (51·5)**
**Urine**[3] (n=2663 recorded)	
Negative/contaminant	1656 (62·2)
Positive microscopy only	118 (4·4)
**Significant positive**[5]	**889 (33·4)**
**Other**[4] (n=86 recorded)	
Negative/contaminant	38 (44·2)
**Significant positive**[5]	**48 (55·8)**

[1]Peripheral and central.
[2]Endotracheal aspirates, bronchoalveolar lavage or pleural fluid.
[3]Urine and catheter specimens of urine.
[4]Other samples include abdominopelvic fluid, high vaginal swabs and miscellaneous pus samples.
[5]Refer to Methods section for definition of significant.

Supplementary Table 3: Cohort characteristics stratified by recording of microbiological investigations

Variable	Microbiological investigations recorded No (n=40,253)	Microbiological investigations recorded Yes (n=8649)	p-value
**Demographics**			
Male sex	22669 (56.3)	5310 (61.4)	<0.001
Age, years, mean (SD)	70.2 (17.8)	68.1 (18.2)	<0.001
**Co-morbidities**			
Chronic cardiac disease	12247 (30.4)	2528 (29.2)	<0.001
Hypertension	8631 (21.4)	2014 (23.3)	0.001
Chronic pulmonary disease	10774 (26.8)	2395 (27.7)	<0.001
Immunocompromise	5993 (14.9)	1555 (18.0)	<0.001
Asthma	5138 (12.8)	1171 (13.5)	<0.001
Chronic kidney disease	6647 (16.5)	1445 (16.7)	<0.001
Chronic liver disease (moderate/severe)	708 (1.8)	159 (1.8)	<0.001
Chronic liver disease (mild)	567 (1.4)	116 (1.3)	<0.001
Chronic neurological disease	4731 (11.8)	1016 (11.7)	<0.001
Current malignancy	3761 (9.3)	969 (11.2)	<0.001
Chronic haematology disease	1523 (3.8)	461 (5.3)	<0.001
HIV/AIDS	143 (0.4)	41 (0.5)	<0.001
Obesity[1]	3782 (9.4)	1095 (12.7)	<0.001
Diabetes mellitus	11 114 (27.7)	2467 (28.5))	<0.001
Rheumatological disease	4081 (10.1)	984 (11.4)	<0.001
Dementia	6385 (15.9)	1149 (13.3)	<0.001
Smoking			
Current	1940 (4.8)	407 (4.7)	<0.001
Never	14458 (35.9)	3495 (40.4)	
Former	8678 (21.6)	2147 (24.8)	
**Pre-admission**			
Symptom onset prior to admission, days, median (IQR)	5.7 (6.9)	6.2 (6.7)	<0.001
Receipt of antimicrobials prior to admission	10836 (26.9)	2554 (29.5)	<0.001
**Organ support**			
Non-invasive ventilation	4937 (12.3)	1818 (21.0)	<0.001
Invasive mechanical ventilation	2547 (6.3)	1694 (19.6)	<0.001
Renal replacement therapy	912 (2.3)	596 (6.9)	<0.001
Vasopressors	1663 (4.1)	1324 (15.3)	<0.001
**Medical management**			
Anti-bacterial	31415 (78.0)	7843 (90.7)	<0.001
Anti-fungal	221 (0.5)	88 (1.0)	<0.001
Corticosteroids	3705 (9.2)	1145 (13.2)	<0.001
**Outcomes**			
Outcome at ≥28 days			
Discharged alive	24062 (59.8)	4757 (55.0)	<0.001
Ongoing hospitalisation	893 (2.2)	293 (3.4)	
Transfer to other facility	2099 (5.2)	524 (6.1)	
Inpatient death	12474 (31.0)	2918 (33.7)	
Palliative discharge	721 (1.8)	148 (1.7)	
Unknown/Awaited	4 (0.0)	9 (0.1)	
Length of stay, days, mean (SD)	12.8 (14.3)	15.8 (15.8)	<0.001
**Vital signs on admission**			
Temperature, °C, mean (SD)	37.3 (1.1)	37.6 (1.1)	<0.001
Respiratory rate, breaths/min, mean (SD)	23.0 (7.0)	24.5 (7.9)	<0.001
Systolic blood pressure, mmHg, mean (SD)	131.4 (25.5)	131.0 (25.5)	0.178
Heart rate, beats/min, mean (SD)	91.7 (21.8)	94.6 (21.9)	<0.001
Oxygen saturation, %, mean (SD)	93.5 (7.7)	92.8 (7.8)	<0.001
**Laboratory results on admission**			
Total white cell count, $x10^9$/L, mean (SD)	8.8 (7.0)	9.1 (7.1)	0.001
Neutrophil count, $x10^9$/L, mean (SD)	7.0 (6.7)	7.1 (5.6)	0.053
Lymphocyte count, $x10^9$/L, mean (SD)	1.5 (5.7)	1.6 (7.0)	0.193
Platelet count, $x10^9$/L, mean (SD)	237.6 (112.1)	234.8 (113.5)	0.039
C-reactive protein, mg/L, mean (SD)	105.9 (94.5)	123.4 (105.7)	<0.001
Procalcitonin, ng/mL, mean (SD)	2.8 (10.0)	3.3 (10.9)	0.104
**Thoracic radiology on admission**			
Thoracic imaging performed on admission	27723 (68.9)	6632 (76.7)	<0.001
Infiltrates present on admission	15795 (39.2)	4321 (50.0)	<0.001

SD, standard deviation; IQR, interquartile range

[1]Clinician defined.

Supplementary Table 4: Deep respiratory microbiology results stratified by sample type

Organism	BAL (n=155)	ETA (n=141)	Other (n=32)
Achromobacter xylosoxidans	1 (0.6)	0	0
Acinetobacter baumannii	0	4 (2.8)	1 (3.1)
Anginosus group streptococci	0	0	3 (9.4)
Aspergillus fumigatus	3 (1.9)	4 (2.8)	0
Bacteroides fragilis	0	0	1 (3.1)
Citrobacter sp.	1 (0.6)	0	1 (3.1)
Citrobacter freundii	3 (1.9)	0	0
Citrobacter koseri	11 (7.1)	11 (7.8)	1 (3.1)
Enterobacter sp.	1 (0.6)	1 (0.7)	0
Enterobacter cloacae	8 (5.2)	8 (5.7)	0
Enterobacter kobei	1 (0.6)	0	0
Enterococcus sp.	2 (1.3)	0	0
Enterococcus faecalis	3 (1.9)	1 (0.7)	1 (3.1)
Enterococcus faecium	2 (1.3)	1 (0.7)	3 (9.4)
Escherichia coli	17 (11)	21 (14.9)	5 (15.6)
Haemophilus sp.	1 (0.6)	0	0
Haemophilus influenzae	3 (1.9)	1 (0.7)	1 (3.1)
Hafnia alvei	1 (0.6)	0	0
Klebsiella sp.	4 (2.6)	0	1 (3.1)
Klebsiella aerogenes	17 (11)	10 (7.1)	1 (3.1)
Klebsiella oxytoca	4 (2.6)	2 (1.4)	0
Klebsiella pneumoniae	15 (9.7)	17 (12.1)	1 (3.1)
Klebsiella variicola	1 (0.6)	0	0
Lactobacillus rhamnosus	0	0	1 (3.1)
Moraxella catarrhalis	1 (0.6)	0	0
Morganella morganii	3 (1.9)	6 (4.3)	0
Proteus mirabilis	5 (3.2)	6 (4.3)	0
Pseudomonas sp.	2 (1.3)	1 (0.7)	1 (3.1)
Pseudomonas aeruginosa	14 (9)	11 (7.8)	2 (6.2)
Serratia sp.	0	1 (0.7)	0
Serratia marcescens	4 (2.6)	5 (3.5)	0
Serratia ureilytica	1 (0.6)	0	0
Staphylococcus aureus	17 (11)	22 (15.6)	4 (12.5)
Stenotrophomonas maltophilia	4 (2.6)	4 (2.8)	1 (3.1)
Streptococcus (Beta-haemolytic)	0	1 (0.7)	0
Streptococcus agalactiae	1 (0.6)	2 (1.4)	0
Streptococcus anginosus	0	0	1 (3.1)
Streptococcus intermedius	0	0	1 (3.1)
Streptococcus pneumoniae	2 (1.3)	0	0
Streptococcus pyogenes	2 (1.3)	1 (0.7)	1 (3.1)

Data are n (%) for each sample type.
BAL: bronchoalveolar lavage, ETA: endotracheal aspirate.
Other included pleural fluid and other samples labelled “deep”.

Supplementary Table 5: Geographical variation in antimicrobial usage

Region	Ward-level			Critical care		
	March	April	May	March	April	May
East of England	1050 (84.8)	1847 (86.2)	361 (79.2)	284 (90.0)	299 (89.2)	35 (92.1)
London	1657 (80.7)	1403 (82.6)	89 (73.1)	593 (91.9)	451 (93.0)	32 (82.1)
Midlands	2698 (79.9)	3620 (81.9)	842 (74.6)	453 (86.5)	522 (88.8)	103 (80.5)
North East & Yorkshire	1069 (79.4)	2612 (79.8)	524 (73.9)	253 (93.4)	387 (86.4)	51 (82.3)
North West	1755 (86.0)	3625 (86.5)	768 (78.7)	312 (93.4)	536 (93.7)	74 (90.2)
South East	1403 (79.9)	2186 (83.9)	4977 (78.0)	407 (91.8)	498 (95.4)	75 (94.2)
South West	679 (82.8)	1030 (79.4)	200 (63.9)	154 (94.5)	182 (97.8)	24 (92.3)
Scotland	354 (72.7)	763 (74.4)	112 (64.4)	150 (87.2)	183 (84.3)	12 (66.7)
Wales	344 (75.1)	337 (79.1)	64 (83.1)	74 (88.1)	99 (90.0)	13 (100.0)

Data are n (%).

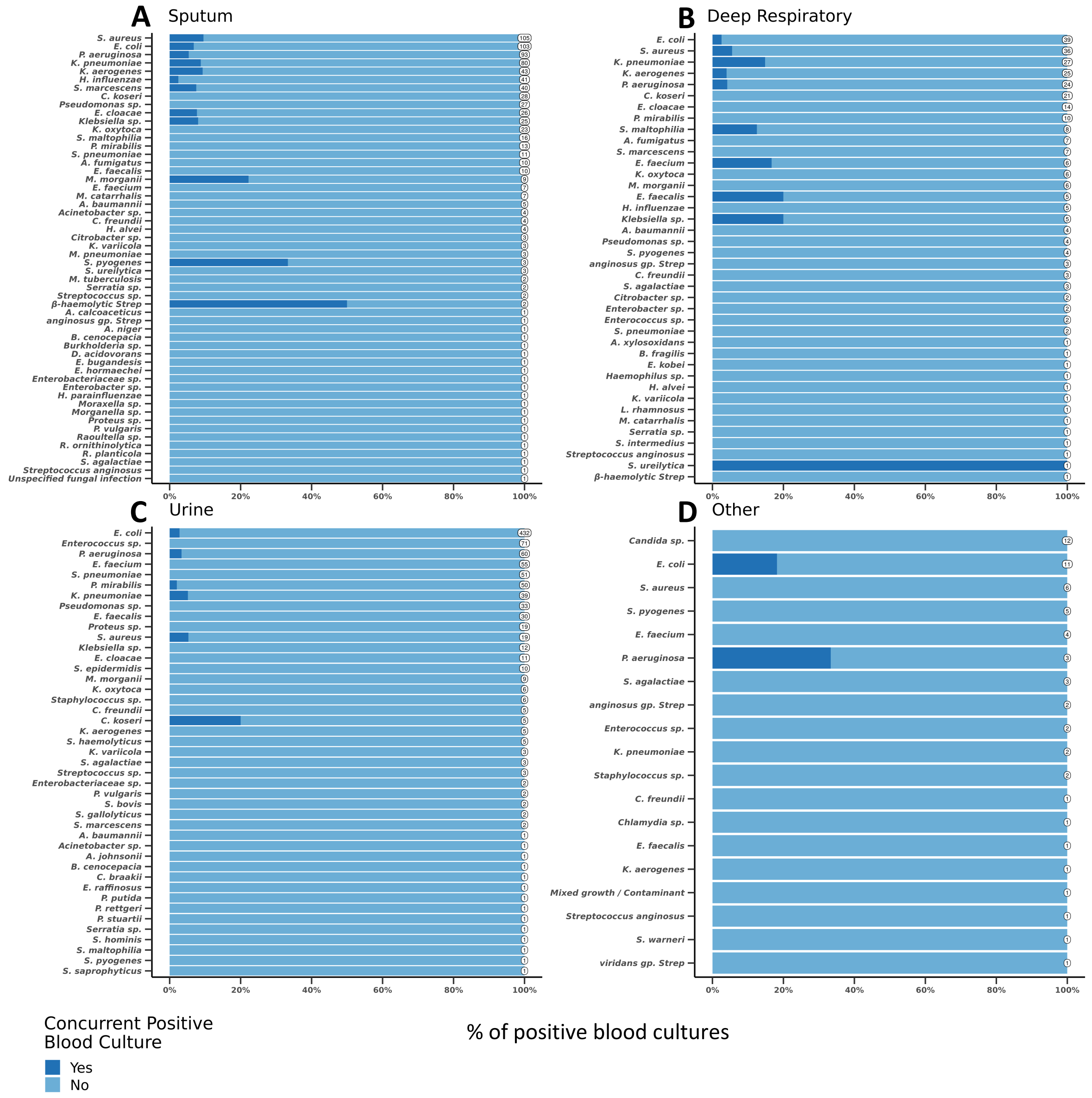

Supplementary Figure 1. Co-detection of pathogens in blood cultures and other sample types

For patients with positive blood cultures, the percentage of instances in which the same pathogen was detected in **(A)** sputum, **(B)** deep respiratory, **(C)** urine or **(D)** other samples is shown. Deep respiratory samples are endotracheal aspirates, bronchoalveolar lavage and pleural fluid. Other samples include abdominopelvic fluid, high vaginal swabs and miscellaneous pus samples. Number at end of bar is the number of times the organism was detected in the listed sample type.

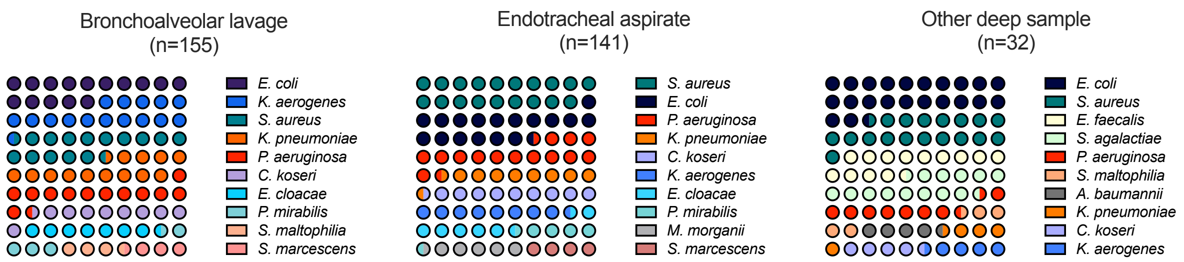

Supplementary Figure 2. Deep respiratory microbiology results stratified by sample type

The 10 most frequently identified organisms from each sample type is shown. The cells in the waffle chart are shaded to represent the total percentage of organisms from that sample type each organism accounts for.

A Critical care

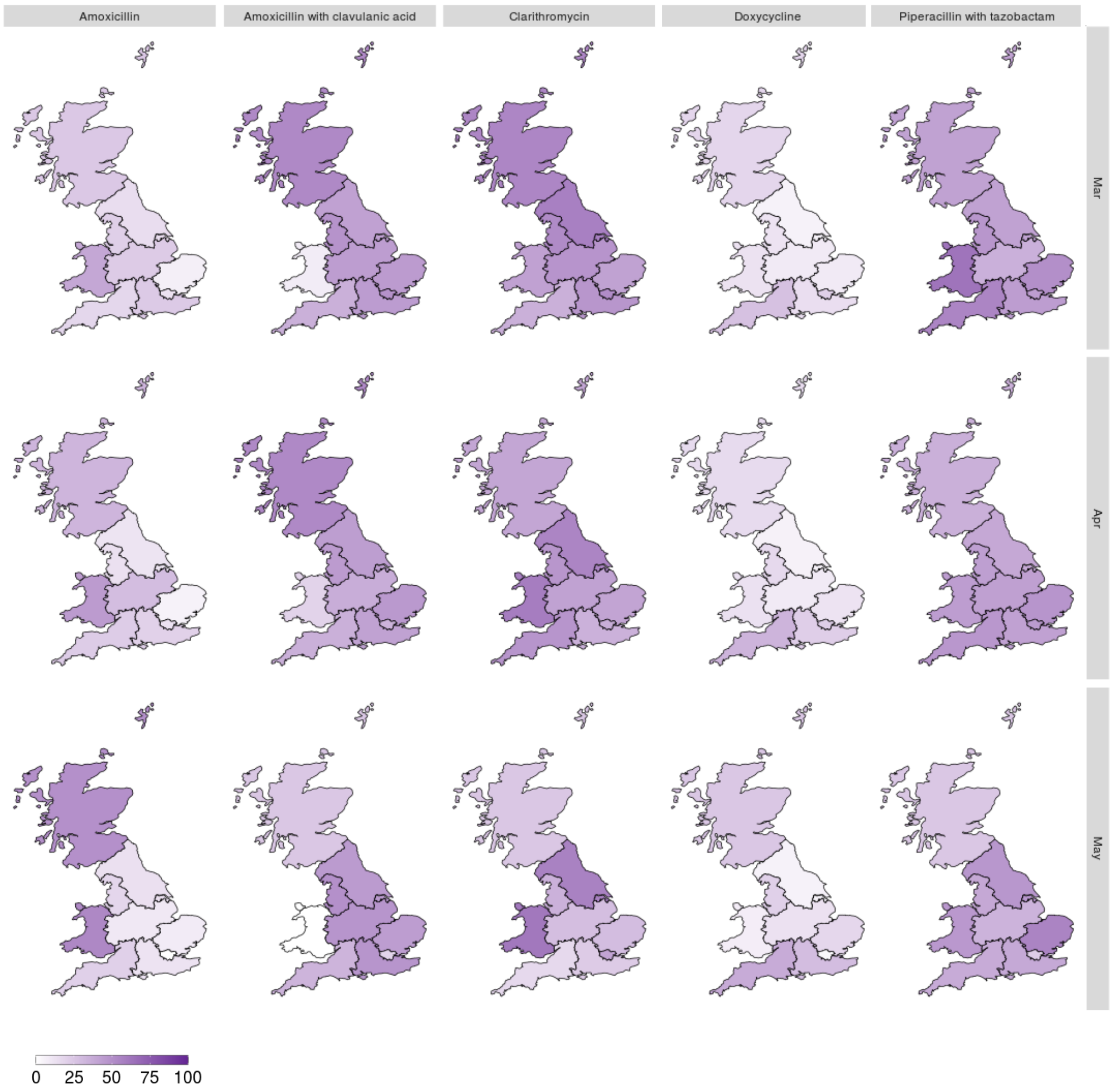

(Legend overleaf)

B Ward-level care

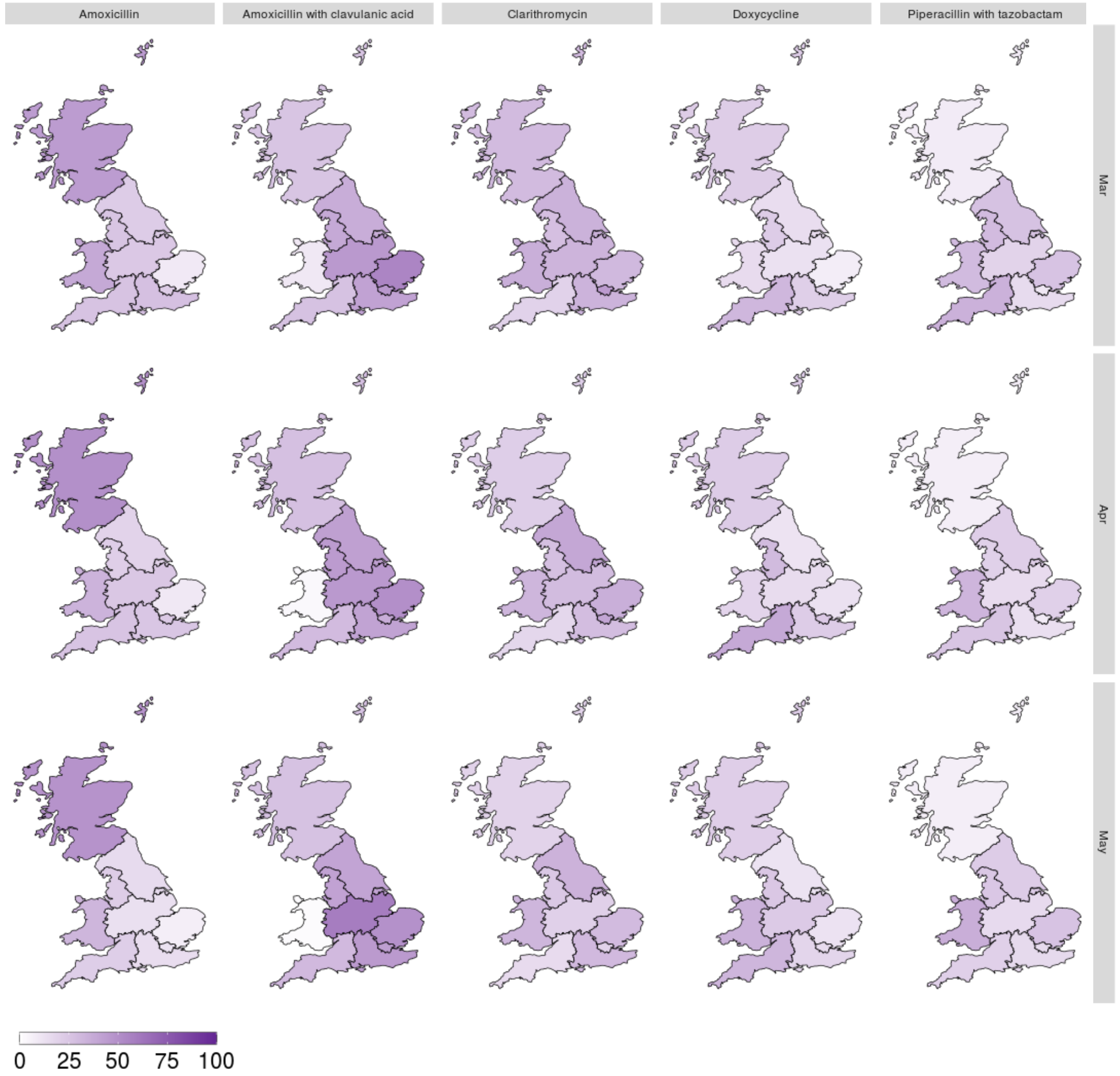

Supplementary Figure 3: Geographical variation in usage of individual antimicrobials over time
Map is divided into Scotland, Wales and regions of England (North East & Yorkshire, North West, Midlands, East, South East, South West, London). Purple shading of regions represents the percentage of patients who received the named antimicrobial during their hospital admission stratified by month of admission (March, April and May). **(A)** shows patients in critical care and **(B)** shows patients receiving ward-level care.

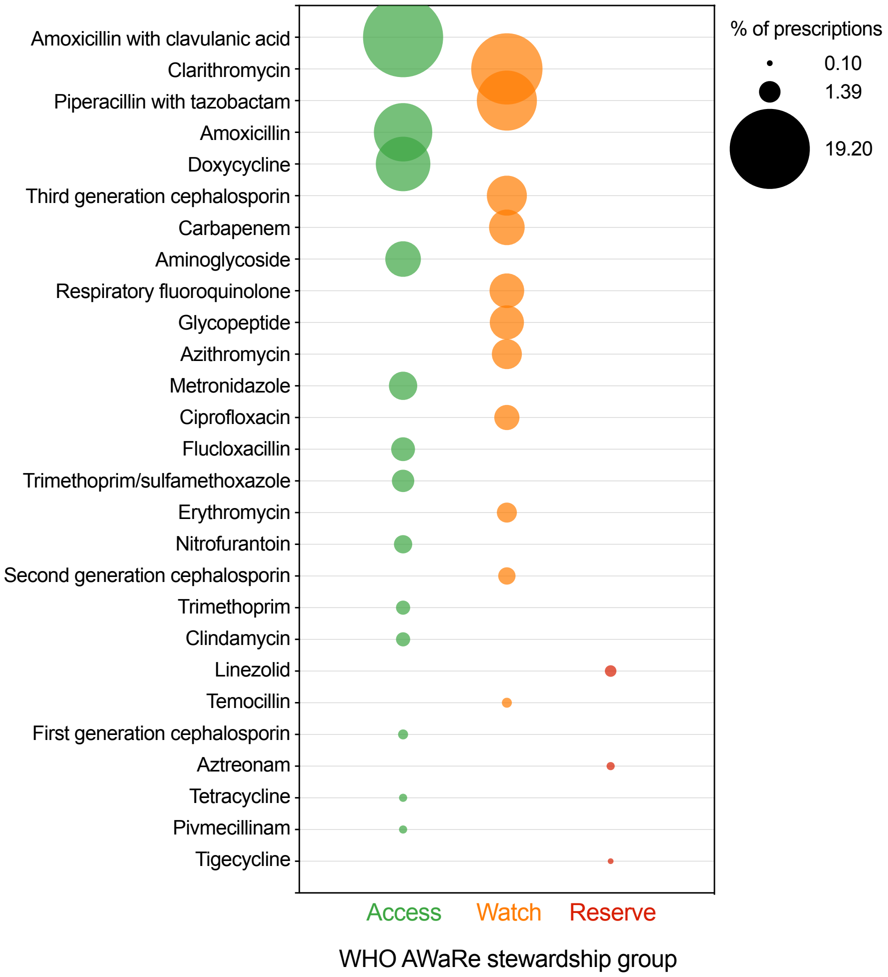

Supplementary Figure 4: Antimicrobial prescriptions classified according to WHO AWaRe classification
The size of each bubble is proportional to the percentage of total prescriptions each antimicrobial accounts for (key to size shown with black bubbles). Bubble are coloured by WHO AWaRe stewardship group (green: "access"; orange: "watch"; red: "reserve"). 50.6% of prescriptions are of "access" antimicrobials, 48.7% are "watch" and 0.7% are "reserve".
